# Controlling Within-Field Sheep Movement Using Virtual Fencing

**DOI:** 10.3390/ani8030031

**Published:** 2018-02-26

**Authors:** Danila Marini, Rick Llewellyn, Sue Belson, Caroline Lee

**Affiliations:** 1 School of Environmental and Rural Science, University of New England, Armidale, NSW 2350, Australia; caroline.lee@csiro.au; 2 CSIRO Locked Bag 2, Glen Osmond, SA 5064, Australia; Rick.Llewellyn@csiro.au; 3 CSIRO Agriculture and Food, Locked Bag 1, Armidale, NSW 2350, Australia; Sue.Belson@csiro.au

**Keywords:** virtual fencing, GPS tracking, animal management, associative learning

## Abstract

**Simple Summary:**

Virtual fencing has the potential to increase the implementation of spatial grazing management and targeted grazing without the use of conventional fencing. Current virtual fencing that uses an algorithm that is patented through CSIRO involves a collar that emits a warning audio when an animal approaches a set boundary. If the animal continues walking towards the boundary, an electric stimulus is applied. Using manually operated collars to implement a similar virtual fence, a small group of sheep were restricted from accessing a section of a small paddock. Sheep were successfully kept out of the excluded zone of the paddock. By the third day, the sheep were able to avoid receiving an electrical stimulus by turning away from the boundary when the warning audio was applied. When the sheep were allowed full access to the paddock again, then they were quick to use the once restricted area.

**Abstract:**

Virtual fencing has the potential to greatly improve livestock movement, grazing efficiency, and land management by farmers; however, relatively little work has been done to test the potential of virtual fencing with sheep. Commercial dog training equipment, comprising of a collar and GPS hand-held unit were used to implement a virtual fence in a commercial setting. Six, 5–6 year-old Merino wethers, which were naïve to virtual fencing were GPS tracked for their use of a paddock (80 × 20 m) throughout the experiment. The virtual fence was effective at preventing a small group of sheep from entering the exclusion zone. The probability of a sheep receiving an electrical stimulus following an audio cue was low (19%), and declined over the testing period. It took an average of eight interactions with the fence for an association to be made between the audio and stimulus cue, with all of the animals responding to the audio alone by the third day. Following the removal of the virtual fence, sheep were willing to cross the previous location of the virtual fence after 30 min of being in the paddock. This is an important aspect in the implementation of virtual fencing as a grazing management tool and further enforces that the sheep in this study were able to associate the audio with the virtual fence and not the physical location itself.

## 1. Introduction

Virtual fencing technology has seen recent rapid advances and demonstrated to be technically feasible and near commercial availability for cattle (agersens.com). The algorithm that is used for cattle was originally developed by the Commonwealth Scientific and Industrial Research Organisation (CSIRO), Australia. The virtual fencing devices use an algorithm that combines GPS with animal behaviour to implement the virtual fence [1,2]. Similar to a conventional fence, virtual fences serve to provide a boundary to contain animals, but unlike conventional fencing, it does not implement a physical barrier [3].

In the case of the virtual fencing system developed by the CSIRO, the animals learn the virtual fence through associating an audio cue with an electrical stimulus. On approach to the fence boundary the audio cue is applied, the stimulus is then only delivered if the animal continues to move forward, if the animal turns away or stops on the audio no stimulus is given. Cattle have been shown to readily learn this association within several approaches [4]; however, there is a high variability in learning and behaviour responses between individuals [5,6]. The potential for virtual fencing to alter the distribution of sheep grazing has been demonstrated, but the understanding and development of virtual fencing technology for sheep is less advanced [7,8,9,10] than for cattle. Research is required to determine the impact that virtual fencing has on sheep welfare, including how it effects normal behavioural patterns.

This experiment looked at the potential use of virtual fencing as a spatial grazing technology for sheep in a commercial setting. Unlike previous studies conducted in rangeland situations [7,8,9], this study investigated the potential application in a crop-livestock (mixed) farming situation where sheep are often used to graze pastures and crop residue in large fenced fields. There is a high level of industry demand for such applications where managing grazing efficiency and animal distribution in large fields could be improved. This is especially the case for fields that contain highly variable and potentially erodible soil types. Managing grazing efficiency can increase profitability, assist natural resource management, and introduces greater potential for targeted intensive grazing to assist weed and cropping management [11]. Electric fencing for grazing management, such as sub-paddock grazing, is rarely used because of the labour and the other costs that are associated with managing temporary fences where farms are often over 3000 ha and fields greater than 150 ha in size. When learning the virtual fence based on location animals may be unwilling to move on from the location they have been trained in. This has been demonstrated previously in cattle where they have been trained to avoid a location using an electrical stimulus alone with no prior auditory warning [12,13]. For virtual fencing to be feasible as temporary fencing for targeted grazing, sheep must be able to cross over areas that were previously fenced off. Therefore, it is important that the animals are able to learn to associate the audio cue with the electrical stimulus.

In this study, the potential for virtual fencing to manage grazing pressure within a field with highly variable soil types was carried out by evaluating the response of a small flock of naïve sheep to the virtual fencing technology in a controlled grazing study deployed on a commercial partner farm. The virtual fence was implemented through the use of manual dog training equipment and applying the associative learning method of linking an audio cue and the animal’s behaviour with an electrical stimulus. The study’s objective was to determine if a group of naïve sheep could be trained to a virtual fence implemented to restrict grazing on erodible sandy soil in the most elevated part of a field. We also aimed to determine if the virtual fence might affect the animals’ normal behaviour as well as their use of the field once the virtual fence was removed.

## 2. Materials and Methods

### 2.1. Ethical Statement

The protocol and conduct of the experiment was approved by the CSIRO Chiswick Animal Ethics Committee under the NSW Animal Research Act, 1985 (approval ARA 17/07).

### 2.2. Equipment

Commercial dog training equipment, which comprises of a collar (Garmin TT15, Garmin Ltd., Olathe, KS, USA) and GPS hand-held unit (Garmin Alpha 100, Garmin Ltd., Olathe, KS, USA) were used. Each collar was paired to its own hand-held unit. When prompted by the hand-held unit, the collars are capable of administering an audio (70–80 dB, 2.7 kHz) and an electrical stimulus, which was set to level 4 (42 mA, 20 us with 16 pulses delivered per s, with no resistance). The stimulus level chosen was based on a pilot trial that was conducted to determine an appropriate electrical stimulus to be used on sheep [10]. Collars were fitted each morning and removed at the end of the day. To record behaviours, the sheep were fitted with HOBO^®^ Pendant G loggers (Onset Computer Corporation, Pocasset, MA, USA), which were fitted and removed each day. The loggers were placed outside of the left hind leg, mid-side on the cannon bone and secured using bandage wraps. The loggers recorded x,y,x coordinates that corresponded to a state of either standing, walking or lying, this was recorded every 2 s between the hours of 9:00 a.m. to 3:00 p.m.

### 2.3. Animals and Experimental Protocol

The trial was conducted on Tapio Station (Wentworth, New South Wales, Australia) located at 34°07′33.7″ S and 142°16′47.5″ E. Six, 5–6 year-old Merino wethers were used in the experiment. For identification, all of the sheep had their flanks marked using sheep branding fluid (Si-Ro-Mark, Cox Agri, County Durham, UK). Throughout the experiment the sheep were kept on crop residue during the day and at night after the tests were moved out of the test paddock and into a holding yard with supplementary hay.

**Figure 1:**
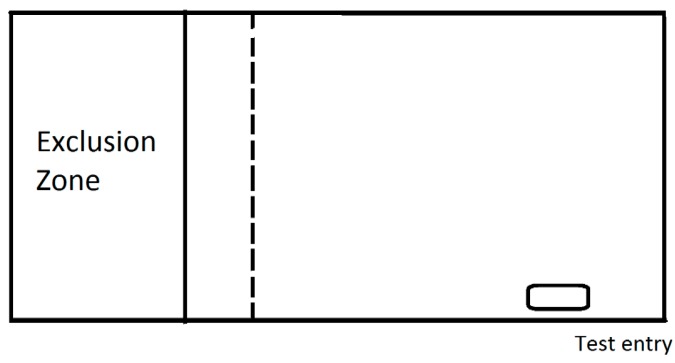
Schematic of the paddock in which sheep were tested in. The small rectangle is the location on the water trough. The warning zone, which is the location in which audios and stimuli were applied is indicated as the dashed line. The solid line is the beginning of the exclusion zone.

Six naive sheep were fitted with Garmin electric dog collars and given complete access to a small paddock (20 m × 80 m) for two days, during which they were tracked by GPS to determine their usage of the paddock. After this, the virtual fence was implemented and animals were prevented from accessing a portion of the paddock for a further three days. The exclusion zone was implemented to prevent access to the top portion of the paddock. This contained a potentially erosion-prone sandy rise, but was where the sheep typically preferred to spend a larger proportion of time (Figure 1; see results Section 3.3). Due to sparse plant cover and the low quality crop residue typical of grazing at the time of year, supplementary feed in the form of lucerne hay was distributed around the paddock including in the exclusion zone. For the final two days of the study, the virtual fence was removed and the sheep were again given access to the whole paddock and their positions continued to be monitored by GPS.

The manual collars were used to specifically implement the CSIRO algorithm. As the sheep entered the warning zone, a 2 s audio cue was delivered. If the sheep displayed either of the following responses: (1) stopping, (2) turning away or backing up, (3) running forward, or (4) moving out of the exclusion zone, the audio cue was ceased before 2 s elapsed. If the sheep failed to respond to the audio cue after 2 s, then an immediate electrical stimulus (maximum 1 s) was applied. If the animal ran towards the exclusion zone, the audio cues and stimulus were not reapplied until the animal had calmed down i.e., stopped running. Once the animal was calm, if they proceeded further into the exclusion zone, the audio cue and a stimulus were reapplied until they turned and exited the exclusion zone. Criteria were included to account for grazing over the warning line. If grazing the sheep were given an audio cue for 2 s, for each step the sheep took forward another audio cue was applied. This was done for a maximum of three audio cues, after the third audio, an electrical stimulus was immediately applied.

Three operators positioned on an elevated viewing platform approximately 20 m outside the boundary of the test paddock were responsible for applying the cues. Each operator controlled the same two sheep each day with a Garmin hand-held unit. The number of audio cues and electrical stimuli, as well as the behavioural response, to the audio and electrical stimulus were recorded by a scribe, and proceedings were videotaped to verify the behaviours.

### 2.4. Statistics

The statistical software package R (The R Development Core Team, Version 3.3.3) packages *nlme* [14] and *pgirmess* [15] was used for analysis. The testing periods were split into three periods for analysis, PreVF = day 1 and 2 prior to implementation of the virtual fence, VF = day 3, 4, and 5 of the test where the virtual fence was implemented, PostVF = day 6 and 7 virtual fence was removed. The number of audio cues and electrical stimulus applied to the animals was analysed using a binomial test to compare within day and Chi-Square test to compare across days. Where appropriate behaviour in response to the audio cue across test days were analysed using proportions of test. Interactions with the fence across the test days was analysed using a repeated measures, linear mixed effects model with animal fitted as a random effect. Data collected from the hobo loggers were aggregated into 15 min intervals with all behaviours summed within the 15 min. They were then collated and presented as an average over the hour. Behavioural data could not be normalised by transformation and were analysed using the Kruskal-Wallis rank sum test to test the effect of test period on behaviour.

For the associative learning test, logistical regression were used to evaluate the learning period in which sheep were able to respond to an audio cue to avoid an electrical stimulus. The logistical regression were fitted to the data using the non-linear least squares function, this method has been previously described [4]. The application of the audio cue and electrical stimulus from each test day was paired to create a binary variable, starting at the first pairing where an audio was followed by a stimulus. The audio event number Xij and a binary variable Zij is zero if the sheep received audio not followed by an electrical stimulus and one if the audio was followed by an electrical stimulus. A general logistic curve of the form of $\pi =a+\frac{c}{1\;+\;\exp\left(\;-b\left(x\;-\;m\right)\right)}$ was fitted where π is the probability that Zi = 1. The upper asymptote = a + c, and is the proportion of naïve animals receiving an electrical stimulus. The lower asymptote = a, and is the proportion of animals that still receive an electrical stimulus after behavioural change. The slope parameter = b, which is related to rate of behavioural change, with a negative slope indicating that the proportion of the animals receiving an electrical stimulus following a fence interaction decreases with repeated interactions. The point of inflection = m, is the number of interactions it takes for half of the animals to change their behaviour. This is the midpoint on the curve between the upper and lower asymptote. No constraints were applied in fitting the curve, allowing for the slope parameter to be greater than zero and the asymptote to be outside the meaningful range of zero to one.

### 2.5. GPS Data

The GPS data obtained from the Garmin collars were collected into a 1 min time step template. For any data points that were missing, it was assumed that the sheep did not move and the missing data was replaced with the previous known GPS location. The paddock the sheep were in was mapped and a ‘vector grid’ and a 5 × 5 cell of the paddock was created. The grid map shows how many minutes each sheep spent in each cell of the paddock over the test period. This was done for each sheep each day, and sheep were combined to create an overall map for the flock.

## 3. Results

### 3.1. Learning the Virtual Fence

During the three days that the virtual fence was implemented animals had a higher percentage of audio cues than electrical stimulus. Within each day, animals were more likely to not receive an electrical stimulus following an audio cue (*p* < 0.05). The proportion of audio to electrical stimulus did not vary significantly over the three days (χ^2^ (2) = 1.49, *p* = 0.47, Table 1).

**Figure 2:**
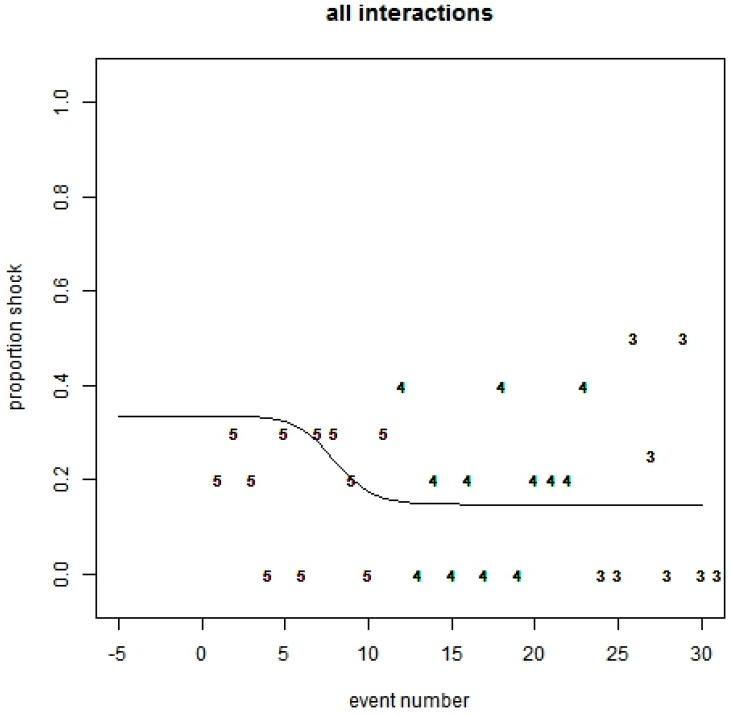
Logistic curve for the associative learning trial for six sheep. Y-Axis is the proportion of animals that receive a stimulus following an audio cue. X-Axis is number of interactions with the virtual fence throughout the entire testing period. The numerals are the number of animals that approached the exclusion zone for that event number.

The logistic model used in previous studies did not fit the data well, as seen by the observed proportions not being close to the fitted line (Figure 2). It took an average of eight interactions for an association to be made. Even so, the probability of a sheep receiving an electrical stimulus following an audio cue is low (19%). The probability of receiving an electrical stimulus before and after learning (significance of slope) is not significant (*p* = 0.65, Table 2).

### 3.2. Behavioural Response to the Virtual Fence

There were not enough instances of individual behaviours to analyse in response to the audio and electrical stimulus. Raw data of these behaviours are presented in Table 3 and Table 4. Over the course of the three days, the proportion turning behaviour in response to the audio increased (χ^2^ (2) = 40, *p* < 0.05) and the proportion of grazing decreased (χ^2^ (2) = 20, *p* < 0.05). By day 3, interactions with the fence decreased; however, this was not significant (*p* = 0.2).

**Figure 3:**
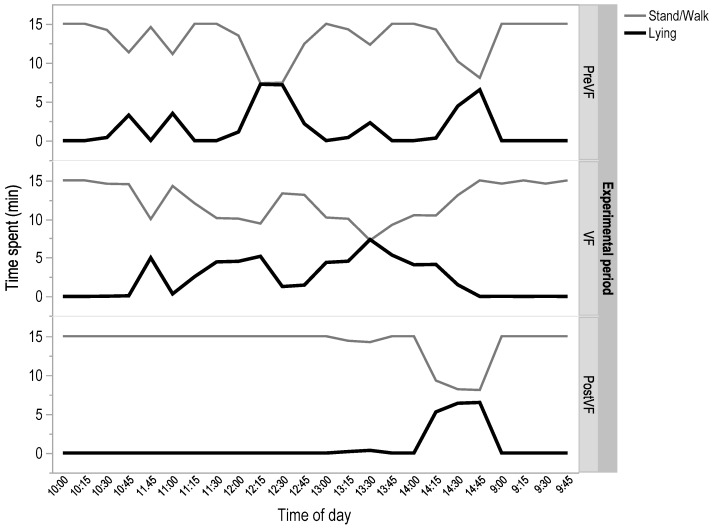
Mean time within 15-min sample intervals that sheep (*n* = 6) spent lying or standing recorded between the hours of 9 a.m. to 3 p.m. PreVF = day 1 and 2 prior to implementation of the virtual fence, VF = day 3, 4, and 5 of the test where the virtual fence was implemented, PostVF = day 6 and 7 where no virtual fence was implemented.

For behavioural time budgets there was a test by time effect seen for walking (H (2) = 30, *p* < 0.05) and lying (H (2) = 20, *p* < 0.05), with lying patterns varying throughout the day (H (5) = 40, *p* = 0.05). Overall the sheep spent a total of 4 h standing before and during the virtual fence test and 5 h standing on the days following the removal of the virtual fence. The times at which behaviours were displayed varied for each test phase. For the average amount of time spent standing in each hour, there was a significant difference between PostVF when compared to the other test phases (difference = 36.03 for PreVF and 28.14 for VF), with a critical difference of 16.5 (α = 0.05). The biggest change was seen in relation to lying (Figure 3) with animals only spending a mean time of 19 mins lying PostVF, as compared to 40 mins to 1 h in the PreVF and VF test, respectively (difference = 21.01 for PreVF and 30.74 for VF), with a critical difference of 16.5 (α = 0.05).

### 3.3. Use of the Paddock

**Figure 4:**
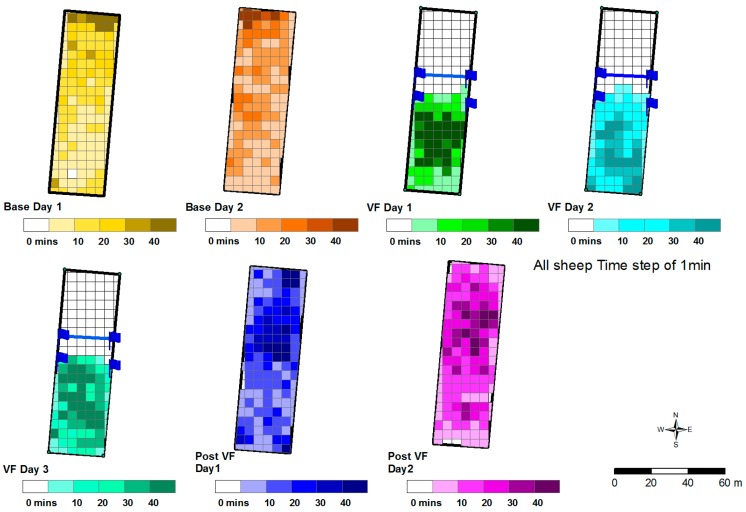

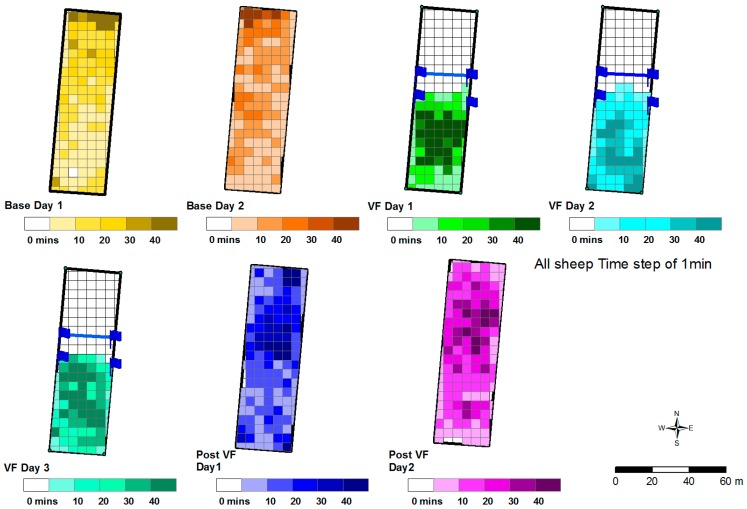
GPS tracking of the six sheep’s use of the paddock throughout the trial. Base Day 1 and 2 is prior to the virtual fence application (Pre VF), VF day 1 to 3 is during the application of the virtual fence and Post VF day 1 and 2 is after the fences removal. The flags with no line between them indicate the start of the warning zone. The flags joined with a line indicate the start of the exclusion zone.

Prior to the implementation of the virtual fence sheep accessed the entirety of the paddock. During the three days the virtual fence was implemented sheep were successfully kept from accessing the top part of the paddock (the exclusion zone). When the virtual fence was removed animals again accessed the entire paddock (Figure 4). It took the sheep 30 min on the first day following the removal of the virtual fence to approach and breach the pre-existing virtual fence line. 

## 4. Discussion

In this study, the use of manual training collars to implement a virtual fence was successful at restricting a small group of sheep within a section of a paddock. The sheep demonstrated that they were able to respond to the audio cue alone after two days in which the virtual fence was implemented. Due to the group interactions, an individual sheep’s probability of receiving a stimulus was quite low even prior to learning to associate the audio cue with the stimulus. Following the removal of the virtual fence, animals were quick to cross the location in order to access the other end of the paddock, indicating that the animals had learnt to respond to the cues rather than the location of the fence. In this study, animals were given auditory warning prior to the application of an electrical stimulus, which was only applied if animals did not turn or stop on the audio. The sheep had a large number of interactions with the fence and were willing to spend time close to the virtual fence location but were still successfully restricted to a portion of the paddock. Baseline GPS data show that the sheep had preferred locations at the top of the paddock, suggesting sheep would have had motivation to try and access this section of the paddock. When the virtual fence was removed the sheep were quick to approach the original virtual fence boundary and cross it when no audio cues or electrical stimulus were given. Results of our study were similar to recent work by Campbell, et al. [16], which looked at implementing a virtual fence with cattle in a similar paddock situation as well as implementing changes in the virtual fences location within the paddock. Cattle were restricted from using 60% down to 20% of the paddock, with the location of the virtual fence changing every few days. The final fence change implemented a fence line to split the paddock lengthways rather than across as had been done previously. The cattle were successfully restricted within their allowed grazing zone during the study, when the fences were moved cattle would cross the old boundary and interact with the new one within 5 h of the fence being relocated [16].

In contrast, the study by Markus, et al. [13], which looked at comparing a normal electrical fence with a virtual fence in restricting cattle’s access to a trough found that cattle trained to the virtual fence were not willing to cross its location following its removal. This study suggested that cattle were wary of the location of where the virtual fence was implemented and that virtual fencing may affect cattle movement behaviour even after it has been removed. The study by Markus, et al. [13] solely implemented the electrical stimulus with no audio, the cattle would have only had visual and spatial cues to associate with the virtual fence. Therefore it is understandable why it why, cattle would be reluctant to cross a location that is associated with a negative stimulus as the only indication that they are going to receive a negative stimulus is from the location itself. In our study, this reaction was not seen in sheep, and not seen in other cattle studies [16], highlighting the importance in the use of audio cues in warning animals of the virtual fences location.

The sheep in this study had a low probability (19%) of receiving an electrical stimulus following an audio cue. This was evident, even prior to learning to associate the audio cue with the electrical stimulus. While it is evident that individuals learnt in this study, it is conducted in a small group setting and so group influence cannot be discounted. The size of the group can also influence individual sheep movements. In large flocks, sheep are known to form subgroups and responses of individual sheep to passive recruitment can be effected by group size [17]. However, the results in this study are not dissimilar to previous studies [4,10,16]. The low probability could be due to a variety of factors including sheep being sensitive and cautious in response to the audio and responding to it on first exposure or due to the application of three audios during grazing in which a stimulus was only applied following the third audio. As the animals had more exposure to the virtual fence over the three days, their reactions to the audio and stimulus changed. Over the course of the three days, the number of animals that turned in response to the audio increased and the number of animals ignoring the audio and continuing to graze decreased. This change in behaviour is indicative of animals learning the association of the audio with the electrical stimulus and reacting appropriately once receiving the audio in order to avoid the stimulus. This is similar to responses seen in cattle trained to a virtual fence. As cattle had more exposure to the audio and stimulus they were more likely to turn in response to the audio [16]. On days 1 and 2 where interactions are comparable animals were less likely to display the exaggerated response of jumping after repeated exposure. Other work in sheep has reported mild reactions in response to the electrical stimulus, but have not reported on whether the sheep’s reaction to the stimulus changes over time [8,9].

Normal behaviour was affected in the two days following removal of the virtual fence in this study, particularly the amount of time spent lying compared to the initial period without virtual fencing in place. The decrease in the amount of time spent lying could have been due to the animals now having access to the entire paddock and increasing their time spent grazing. Pasture availability has been shown to affect the amount of time an animal spent grazing [18,19]. A study in cattle looking at the effect of pasture availability found that as pasture biomass decreased, cattle increased their time budget for grazing and walking [20]. Although, pasture biomass was not measured in this study sheep did require supplemental feeding throughout the experiment due to low pasture availability. The removal of the fence gave sheep access to extra feed after being restricted for three days and potentially increasing their motivation to graze. The study by Campbell, et al. [16] found that cattle behaviour was not significantly affected by the implementation of the virtual fence, however, the number of lying bouts was effected. Cattle still displayed similar behavioural patterns throughout the entire testing period. This difference may be due to the cattle having a much longer acclimation period to the paddock and longer testing period compared to the sheep in this study.

## 5. Conclusions

Sheep in this study were able to associate the audio with the virtual fence and not the physical location itself. Virtual fencing can successfully exclude a small group of sheep from an area within a field. Once the virtual fence is removed the sheep rapidly return to grazing the full field. The sheep’s normal behaviour was shown to be affected by the implementation and removal of the virtual fence in this study. Longer term studies need to be conducted to determine the effect of virtual fencing on normal patterns of animal behaviour. The results highlight the potential for virtual fencing of sheep to be used for improved grazing and natural resource management; however, more research needs to be conducted on a larger scale or with more replication, as sheep may respond differently in larger groups. In order to better understand potential commercial application, future research also needs to investigate the potential for managing sheep movement when only a proportion of sheep are wearing a virtual fencing device.

## Figures and Tables

**Table 1:** Contingency table of the number of times the electrical stimulus was or was not applied to six sheep, following an audio cue during the virtual fence (VF) test.

VF Day		No Electrical Stimulus	Electrical Stimulus
1	Count	52	14
	Percentage	78.79%	21.21%
	Std Residual	−0.29	0.64
2	Count	50	11
	Percentage	81.97%	18.03%
	Std Residual	0.42	0.02
3	Count	31	4
	Percentage	88.57%	11.43%
	Std Residual	0.42	−0.91
	Count total	133	29

**Table 2:** Estimated parameters for the logistic regression curves for all training days. The upper asymptote indicates the proportion of naïve animals that received a stimulus during these events. The lower asymptote is the proportion of animals that continued to receive a stimulus. The difference between these is tested for significance. The point of inflection is the mean number of attempts it takes for half of the learning to occur. The slope indicates the speed of transition from the upper to lower asymptote.

Upper Asymptote	Lower Asymptote	Significance of Difference	Point of Inflection	Slope	Significance of Slope
0.33	0.19	0.07	7.9	−0.89	0.65

**Table 3:** Count of behaviours from six sheep, displayed in response to the audio cues over the three days the virtual fence was implemented.

Response to Audio	Day 1	Day 2	Day 3
Continue forward	1	0	0
Turn	5	13	23
Stop	9	1	0
Grazing	37	34	8
Flinch	0	2	0
Total interactions	52	50	31

**Table 4:** Count of behaviours from six sheep, displayed in response to the electrical stimulus over the three days the virtual fence was implemented.

Response to Electrical Stimulus	Day 1	Day 2	Day 3
Turn	2	4	1
Jump	9	4	3
Flinch	1	1	0
Stop	2	0	0
No reaction	0	2	0
Total interactions	14	11	4
